# Challenges in Diagnosis and Management: A Case of Abdominal Wall Liposarcoma

**DOI:** 10.7759/cureus.67643

**Published:** 2024-08-23

**Authors:** Ashish Jivani, Raju K Shinde, Bhushan Jajoo

**Affiliations:** 1 General Surgery, Jawaharlal Nehru Medical College, Datta Meghe Institute of Higher Education and Research, Wardha, IND; 2 Surgical Oncology, SGM cancer hospital, Wardha, IND; 3 Surgical Oncology, Jawaharlal Nehru Medical College, Datta Meghe Institute of Higher Education and Research, Wardha, IND

**Keywords:** chemotherapy, flap reconstruction, surgical resection, retroperitoneal tumor, abdominal wall, dedifferentiated liposarcoma

## Abstract

This case report presents the rare occurrence of a large dedifferentiated liposarcoma originating from the abdominal wall in a 57-year-old male. The patient's initial complaint was the gradual development of an abdominal mass over six months without associated urinary or digestive symptoms. Clinical examination revealed a firm, non-mobile mass in the left lumbar region, prompting further investigation. Imaging studies confirmed the presence of a sizable soft tissue mass with calcifications, suggestive of a sarcoma. Preoperative biopsy indicated a malignant mesenchymal tumor, leading to surgical intervention. Intraoperative findings revealed characteristics consistent with a low-grade malignancy, prompting complete tumor resection with flap reconstruction. Subsequent histopathological analysis confirmed the diagnosis of dedifferentiated liposarcoma with negative surgical margins. The patient was referred for palliative chemotherapy due to the aggressive nature of the tumor. This case underscores the diagnostic challenges and therapeutic considerations associated with rare abdominal wall liposarcomas, emphasizing the importance of a multidisciplinary approach in their management.

## Introduction

Abdominal wall liposarcomas are rare neoplasms that arise from adipose tissue within the abdominal wall and represent a small subset of soft tissue sarcomas. They account for approximately 5%-20% of all soft tissue sarcomas, with most cases occurring in adults aged 50-70 [1]. Liposarcomas are classified into four histological subtypes: well-differentiated, dedifferentiated, myxoid/round cell, and pleomorphic. Dedifferentiated liposarcomas, in particular, are characterized by well-differentiated liposarcoma adjacent to areas of non-lipogenic sarcoma, representing a higher-grade component [2]. Clinically, abdominal wall liposarcomas often present as painless, slow-growing masses that may become symptomatic as they increase, causing local compression or invasion of surrounding structures. Diagnosis is typically confirmed through imaging studies, including computed tomography (CT) or magnetic resonance imaging (MRI), which demonstrate the extent of the tumor and aid in surgical planning [3]. A preoperative biopsy may be performed to establish a histological diagnosis and guide treatment strategies [4].

Surgical resection with wide margins remains the primary treatment modality for abdominal wall liposarcomas, aiming to achieve complete tumor excision while preserving surrounding structures and function. Reconstruction following resection may be necessary, particularly in cases where primary closure is not feasible [5]. Adjuvant therapies, such as chemotherapy and radiation, may be considered based on tumor grade, size, and risk of recurrence [6]. Despite advancements in diagnosis and treatment, dedifferentiated liposarcomas pose significant challenges due to their aggressive behavior and propensity for local recurrence and metastasis. Thus, a multidisciplinary approach involving surgical oncologists, radiologists, pathologists, and medical oncologists is essential to optimize patient outcomes and improve survival rates [7].

## Case presentation

A 57-year-old man presented with a six-month history of an enlarging mass in his abdomen. He had noticed the mass gradually increasing in size without any associated urinary or digestive symptoms. The patient had no significant medical history, including no prior therapeutic irradiation or familial history of similar conditions. Upon examination, a palpable mass measuring 12 x 7 cm was identified in the left lumbar region. The mass felt firm and had well-defined borders, with no mobility observed during respiration. Notably, the mass did not cross the midline and was intra-abdominal, as demonstrated by the cough test. There were no signs of intrinsic mobility or evidence of organomegaly. Routine blood investigations, including complete blood counts, liver function tests, random blood sugar, and coagulation profiles, were all within normal limits.

**Figure 1:**
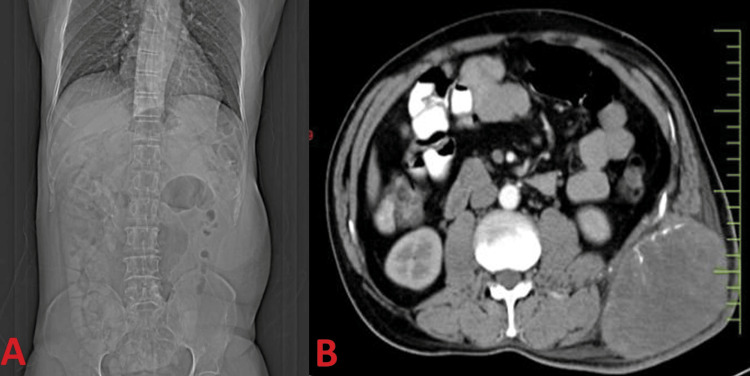
A) Shows a bulge in the left flank and B) shows a retroperitoneal soft tissue sarcoma

Further imaging investigations were pursued, including an abdominal-pelvic CECT scan, which revealed a large, heterogeneously enhancing soft tissue mass within the left lumbar subcutaneous fat. The mass measured approximately 9.8 x 7.7 x 10 cm and exhibited foci of linear calcifications, suggesting a sarcoma. Notably, no intra-abdominal or intercostal extension was noted, and there was no involvement of underlying bone or ribs (Figure 1A, 1B).

Given the suspicion of a malignant mesenchymal tumor based on imaging findings, a preoperative biopsy was performed, confirming the presence of a low-grade malignant mesenchymal tumor. Subsequently, the patient was scheduled for surgical intervention, aiming for complete tumor resection. Frozen sections were obtained intraoperatively, revealing characteristics consistent with a low-grade malignancy. Consequently, a complete resection of the tumor was performed, along with the involved skin. However, primary closure was not feasible, necessitating reconstruction.

**Figure 2:**
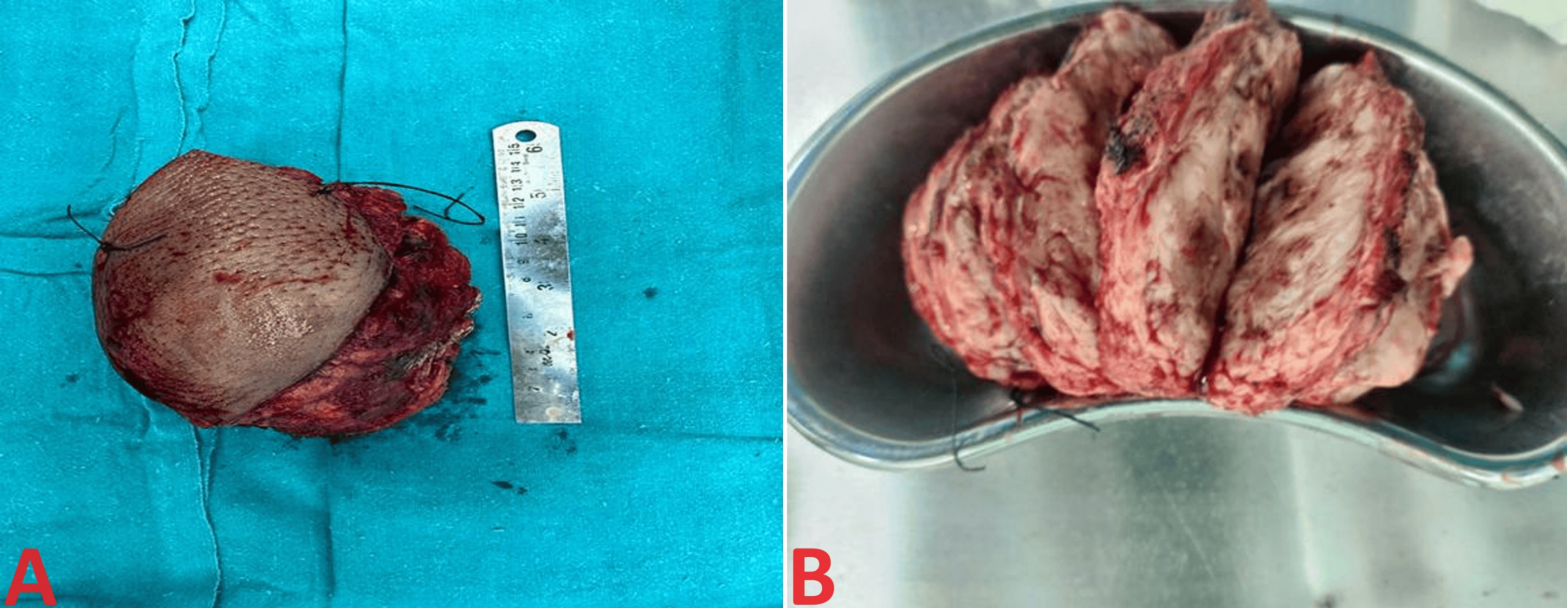
A) Macroscopic appearance of a voluminous dedifferentiated liposarcoma, the retro-peritoneal mass of 14.5 x 11.5 x 8.5 cm firm and whitish mass with skin flap measuring 11 x 8 cm. B) Cut section appears gritty, cystic areas filled with mucoid material seen

**Figure 3:**
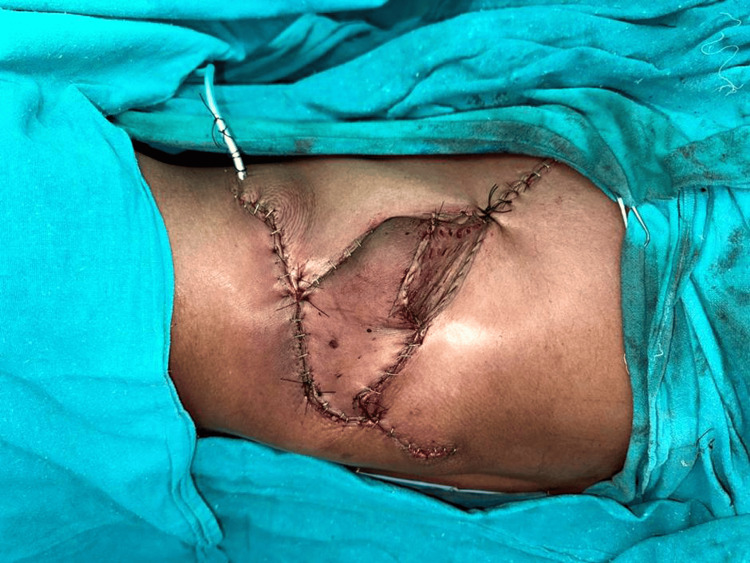
Illustrative image of wound closure with propeller flap repair and drains in place on immediate post-operatively

A propeller flap based on the posterior intercostal artery perforator flap was raised for reconstruction and transposed over the resected site, as shown in the macroscopic appearance in Figure 2. Postoperative histopathological analysis of the resected specimen revealed dedifferentiated liposarcoma, with macroscopic examination showing a yellow-white, firm mass with cystic areas filled with mucoid material. Importantly, all surgical margins were negative for malignant cells. An illustrative image of wound closure with propeller flap repair and sutured flap has been depicted in Figure 3. 

**Figure 4:**
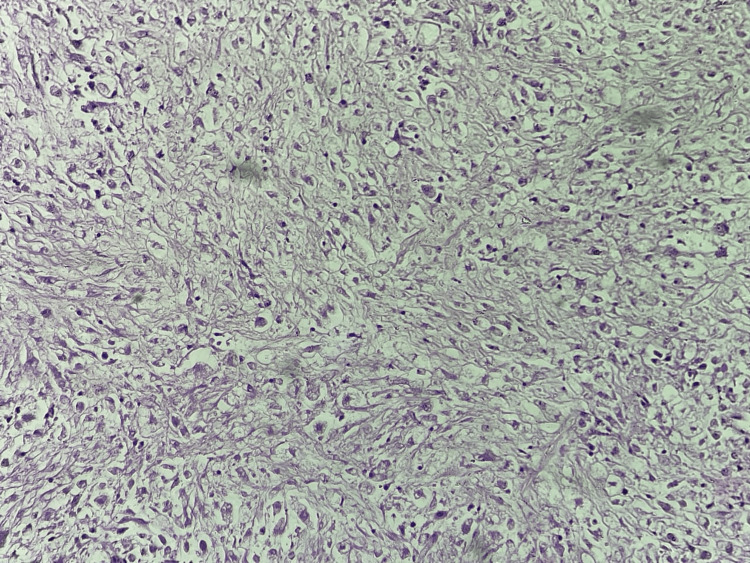
Histopathological examination shows the dedifferentiated liposarcoma images

**Figure 5:**
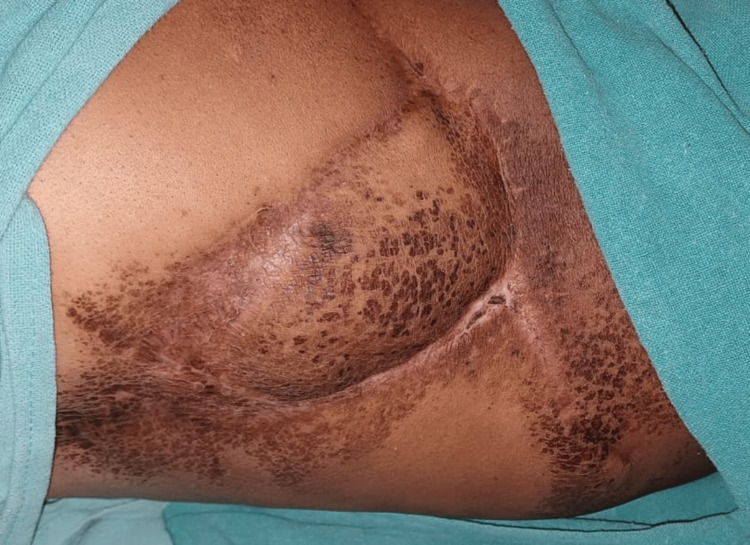
Follow-up image of the patient

Following the histopathological diagnosis (Figure 4), the patient's case was discussed with the medical oncologist, who recommended palliative chemotherapy, given the aggressive nature of dedifferentiated liposarcomas. The patient was subsequently referred for further management and treatment. The follow-up image of the patient is shown in Figure 5.

## Discussion

The presented case highlights several important clinical and therapeutic considerations in managing dedifferentiated abdominal wall liposarcoma. Liposarcomas are a heterogeneous group of soft tissue tumors. Dedifferentiated liposarcomas represent a particularly aggressive subtype, characterized by the presence of non-lipogenic sarcomatous areas juxtaposed with well-differentiated liposarcoma [8]. Abdominal wall liposarcomas are rare entities, comprising a small fraction of all soft tissue sarcomas, and their diagnosis and management pose significant challenges due to their atypical presentation and proximity to vital structures [9].

Surgical resection remains the cornerstone of treatment for localized liposarcomas, aiming for complete excision with negative surgical margins to minimize the risk of recurrence and improve patient outcomes [10]. In the presented case, complete tumor resection was achieved, supported by intraoperative frozen sections suggestive of a low-grade malignancy. However, due to the size and location of the tumor, primary closure was not feasible, necessitating flap reconstruction to optimize wound healing and functional outcomes [11].

Histopathological analysis of the resected specimen confirmed the diagnosis of dedifferentiated liposarcoma, characterized by well-differentiated liposarcoma components alongside non-lipogenic sarcomatous areas. This histological subtype is associated with a high risk of local recurrence and distant metastasis, underscoring the importance of adjuvant therapies in managing dedifferentiated liposarcomas [12]. While the role of adjuvant chemotherapy and radiation therapy remains controversial, recent studies have suggested potential benefits in improving local control and overall survival, particularly in high-grade and unresectable tumors [3].

In the presented case, the decision to pursue palliative chemotherapy was made in consultation with the medical oncologist, considering the aggressive nature of dedifferentiated liposarcomas and the high risk of recurrence and metastasis. Palliative chemotherapy controls disease progression, alleviates symptoms, and improves quality of life in advanced or metastatic disease settings [13]. However, the efficacy of chemotherapy in dedifferentiated liposarcomas remains limited, highlighting the need for further research to identify novel targeted therapies and improve treatment outcomes [14].

## Conclusions

In conclusion, the presented case underscores the challenges inherent in diagnosing and managing dedifferentiated abdominal wall liposarcoma, a rare and aggressive soft tissue tumor. Through a multidisciplinary approach involving surgical intervention, histopathological analysis, and consultation with medical oncologists, the patient underwent successful tumor resection with flap reconstruction. However, the diagnosis of dedifferentiated liposarcoma, confirmed by histopathology, necessitated further consideration of adjuvant therapies, highlighting the importance of ongoing research to optimize treatment strategies and improve patient outcomes. Despite the rarity and complexity of abdominal wall liposarcomas, this case emphasizes collaboration among healthcare professionals in navigating the diagnostic and therapeutic challenges of such uncommon malignancies.
